# Insight into the Alcohol-Free Ring-Opening Polymerization of TMC Catalyzed by TBD

**DOI:** 10.3390/polym13101589

**Published:** 2021-05-14

**Authors:** Fabrice Azemar, Olinda Gimello, Julien Pinaud, Jean-Jacques Robin, Sophie Monge

**Affiliations:** ICGM, Univ. Montpellier, CNRS, ENSCM, 34095 Montpellier, France; fabrice.azemar@univ-ubs.fr (F.A.); olinda.gimello@enscm.fr (O.G.); julien.pinaud@umontpellier.fr (J.P.); sophie.monge@umontpellier.fr (S.M.)

**Keywords:** macrocycles, ring opening, organocatalysis, zwitterionic, trimethylene carbonate

## Abstract

We report herein a study on the alcohol-free, ring-opening polymerization of trimethylene carbonate (TMC) in THF, catalyzed by 1,5,7-triazabicyclo [4.4.0] ec-5-ene (TBD) with ratios n_TBD_/n_TMC_ ranging between 1/20 and 1/400. In all cases, the reaction proceeds very rapidly, even faster than in the presence of alcohol initiators, and provides PTMC with molecular weights up to M_n_ = 34,000 g mol^−1^. Characterization of the obtained PTMC samples by MALDI-TOF mass spectrometry, triple detection size exclusion chromatography and ^1^H NMR spectroscopy reveals the presence of both linear and cyclic polymer chains.

## 1. Introduction

Aliphatic polycarbonates are an interesting class of biocompatible and biodegradable polymers that find applications in the biomedical field for tissue engineering, hydrogel production and drug delivery [1]. Although they were described a long time ago by Carothers [2], they have received increased attention in recent years with the development of functional monomers governing the properties and microstructures of the resulting polymers [3,4,5,6]. This class of polycondensates can be synthesized by usual step growth polymerization, polyaddition of carbon dioxide onto polyepoxides [7] or by ring-opening polymerization (ROP) of cyclic carbonates according to various mechanisms. These different pathways have been reviewed by Mespouille [8] and Feng [9]. Among the various well-known chemical structures of cyclic carbonates [8,10], trimethylene carbonate (TMC) certainly stands out by its commercial availability. Its ROP leads to poly(trimethylene carbonate) (PTMC), a low glass transition temperature Tg (Tg ≈ −20 °C) polymer that has been extensively studied in terms of degradation with different results observed between in vivo [11] and in vitro [12,13] conditions. In particular, quick surface erosion was observed in vivo depending on polymer molecular weights and enzyme action. Beyond PTMC homopolymers, researchers investigated architectures such as stat or block copolymers [14,15,16,17,18] and graft ones from natural polymers [19,20] mainly in view of biomedical applications [12,21,22,23,24,25] where various properties were observed, such as thermo-reversible gelation, self-assembly or hydrogel formation.

Among all the catalytic systems reported in the literature for the ROP of TMC [8], Brønsted and Lewis base catalysts, such as 1,5,7-triazabicyclo-[4.4.0] dec-5-ene (TBD), 1,8-diazabicycloundec-7-ene (DBU) or N-heterocyclic carbenes (NHCs) may represent the most active systems, particularly when combined with a thiourea co-catalyst [26]. They also allow the best control of the polymerization, particularly 1,5,7-triazabicyclo [4.4.0] dec-5-ene (TBD) that proved effective for the preparation of both oligomers [27] and high molecular weight polycarbonates [28]. In both cases, polymers with Ð around 1.5–1.6 were obtained in bulk or in solution using alcohol initiators [28]. Consequently, ROP of TMC or TMC derivatives using TBD as catalyst has become the preferred choice for the production of PTMC based materials. Interestingly, while the ROP of TMC catalyzed by TBD and initiated by alcoholic species predominates in the recent literature, no study has been conducted on the alcohol-free ROP of TMC with TBD. The latter may be of particular interest for the production of macrocyclic PTMCs since other strong organic bases such as NHCs [29] and DBU [30] have already proven effective for the alcohol-free zwitterionic ROP (ZROP) of other cyclic carbonates leading to macrocyclic species.

Therefore, in the present article, we wish to report a study on the alcohol-free ROP of TMC catalyzed by TBD. Particular attention is directed towards the determination of the structure of generated polymers. The latter was achieved through the use of a combination of techniques, including ^1^H NMR, MALDI-TOF mass spectrometry and triple detection size exclusion chromatography. Moreover, polymerization kinetics in the presence/absence of alcohol initiator were also studied to provide further evidence on the mechanism occurring during the TBD-catalyzed ROP of TMC.

## 2. Materials and Methods

### 2.1. Materials

Trimethylene carbonate (TMC) was purchased from TCI (Zwijndrecht, Belgium) and was kept under anhydrous atmosphere after recrystallization twice in dry tetrahydrofuran and drying over high vacuum. Anhydrous tetrahydrofuran (THF), toluene, 1,5,7-triazabicyclo [4.4.0] dec-5-ene (TBD), 4-(dimethylamino) pyridine (DMAP), butan-1-ol and benzyl alcohol were purchased from Sigma-Aldrich (Darmstadt, Germany). Catalysts and initiator were distilled or sublimated and kept under nitrogen atmosphere in an appropriate solvent.

### 2.2. Syntheses

Synthesis of poly (trimethylene carbonate) macrocycle. TBD (68 mg, 0.49 mmol) and trimethylene carbonate (1 g, 9.72 mmol) were weighed into a Schlenk tube. The tube was put under reduced pressure for 30 min and then placed under nitrogen atmosphere. 5 mL of anhydrous THF (5 mL) was added, and the homogeneous solution was stirred at room temperature. Conversion was determined by ^1^H NMR spectroscopy (>90%). The reaction was quenched upon addition of benzoic acid, and the polymer was recovered and purified by precipitation in cold diethyl ether. ^1^H NMR (CDCl_3_, 300 MHz): δ 2.0 ppm (m, CH_2_CH_2_CH_2_) and δ 4.2 ppm (OCOCH_2_).

Synthesis of linear poly(trimethylene carbonate). DMAP or TBD (0.10 mmol) and benzyl alcohol (0.10 mmol) in toluene solution were added in a Schlenk tube under nitrogen atmosphere. The solution was heated at 130 °C for the DMAP or kept at room temperature for TBD. Finally, TMC (4.90 mmol) diluted in toluene was added. Conversion was determined by ^1^H NMR spectroscopy (>80%). The solution was concentrated and diluted in a small amount of dichloromethane. The polymer was recovered by precipitation in cold diethyl ether. ^1^H NMR (CDCl_3_, 300 MHz): δ 2.0 ppm (m, CH_2_CH_2_CH_2_), δ 4.2 ppm (OCO*C*H_2_), δ 5.0 ppm (s, OCH_2_C_6_H_5_) and δ 7.0–8.0 ppm (m, CH_2_C_6_H_5_).

### 2.3. Characterizations

Nuclear magnetic resonance (NMR). Bruker Advance DRX 300 (300 Hz) was used to record ^1^H NMR spectra in CDCl_3_ as deuterated solvent (purchased from Eurisotop). Monomer conversions of TMC were calculated by comparing the relative intensity of the peak at 4.4 ppm (CH_2_CH_2_CH_2_) of the monomer to the signal at 4.2 ppm (CH_2_CH_2_CH_2_) of the polymer.

Triple detection size exclusion chromatography (SEC). Variant 390-LC comprising an auto-injector and a guard column (ResiPore, 50 × 7.5 mm) followed by two linear columns (ResiPore, 300 × 7.5 mm, 3 mm particle size) was employed. This instrument contains the following three detectors: a dual-angle (15° and 90°) light-scattering detector, four-capillary bridge viscometer and differential refractive index detector. DMF (+0.1 wt% LiBr) was used as eluent with a flow rate of 0.8 mL min^−1^. The column temperature was set to 60 °C. Data acquisition and calculations were performed using Cirrus Multi GPC/SEC software.

MALDI-TOF mass spectrometry. Analyses were performed on a MALDI-TOF/TOF Bruker Ultraflex III mass spectrometer using a nitrogen laser for MALDI (λ 337 nm). Mass spectra of 2500 shots were accumulated for the spectra at 25 kV acceleration voltage and reflectron lens potentials at 26.3 kV. A mixture of peptides was used for external calibration. Polymer samples were dissolved in dichloromethane at a concentration of 5 mg mL^−1^. The cationization agent used was NaI dissolved in MeOH at a concentration of 10 mg mL^−1^. The matrix used was dithranol and was dissolved in dichloromethane at a concentration of 10 mg mL^−1^. Solutions of matrix, salt and polymer were mixed in a volume ratio of 3:1:1, respectively. The mixed solution was hand-spotted on a MALDI target and left to dry.

## 3. Results and Discussion

**Scheme 1:**
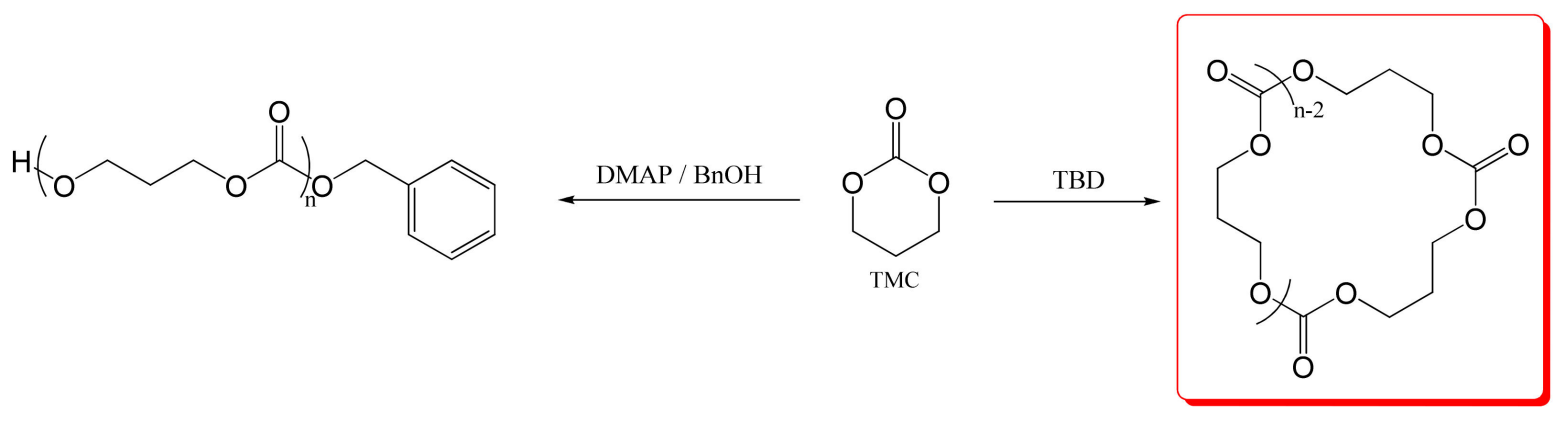
Ring-opening polymerization of trimethylene carbonate (TMC) catalyzed by 1,5,7-triazabicyclo [4.4.0] dec-5-ene (TBD) and by 4-(dimethylamino)pyridine (DMAP)/benzyl alcohol.

Ring-opening polymerization of cyclic esters catalyzed by nucleophilic organic catalysts can proceed by different mechanisms and subsequently lead to polyesters with various macromolecular architectures [31]. In the presence of alcohol initiators, both chain-end and monomer activated mechanisms have been reported to produce linear polymers. In the absence of a protic initiator, the reaction is expected to lead to macrocycles by zwiterrionic ring-opening polymerization (ZROP). Polymerization of trimethylene carbonate (TMC) should proceed similarly (Scheme 1).

**Figure 1:**
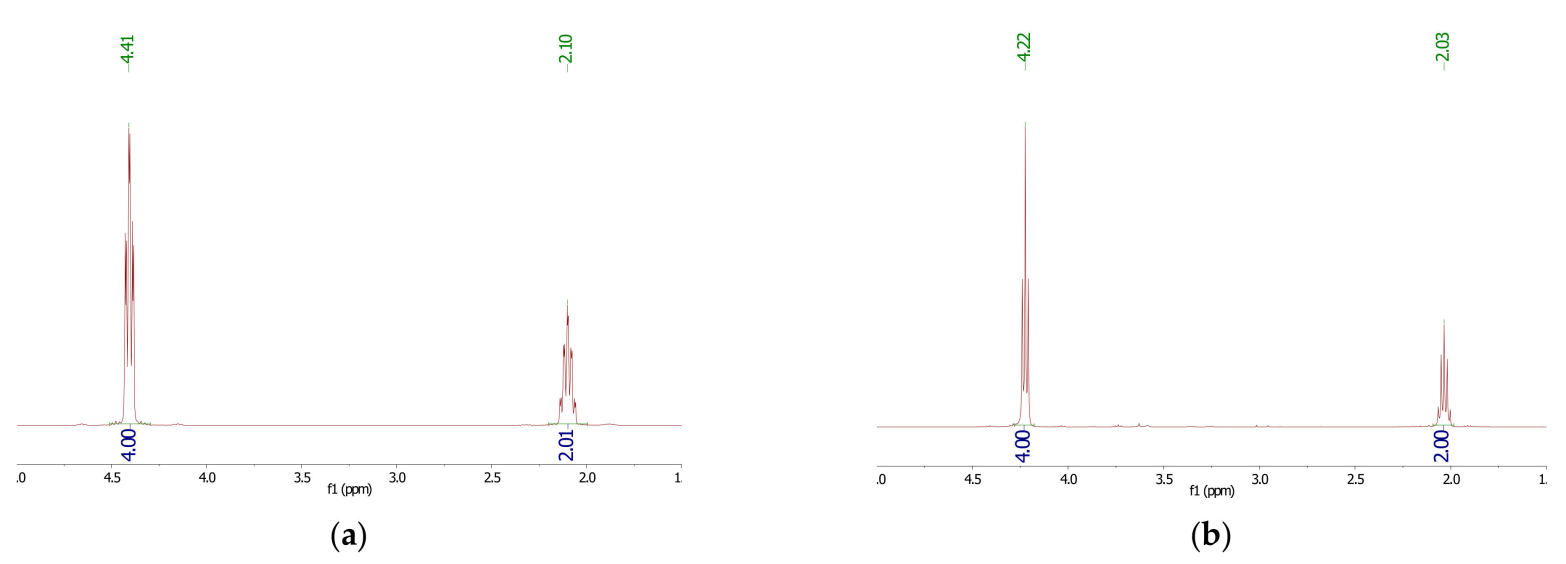
(**a**) ^1^H NMR spectrum of trimethylene carbonate in CDCl_3_ and (**b**) ^1^H NMR spectrum in CDCl_3_ of poly (trimethylene carbonate) PTMC from Exp. 1, Table 1, obtained after precipitation in diethyl ether.

In the first set of experiments, the polymerization of trimethylene carbonate (TMC) was carried out in THF at room temperature (25 °C) without alcohol initiator, using only TBD as catalyst (Exp. 1–4, Table 1). Molar ratios of nTBD/nTMC ranging between 1/20 and 1/400 were employed, and their influences on polymerization kinetics and final polymer molecular weights were evaluated. All reactions were quenched with benzoic acid and samples were analyzed by ^1^H NMR to calculate final conversion of the polymerization and by triple detection size exclusion chromatography (triple SEC) to determine the effective molecular weight (Mn) and molecular weight distribution (Ð) of resulting PTMCs. According to the ^1^H NMR spectra (Figure 1), the ROP of TMC in the presence of TBD only is almost quantitative in the conditions employed (monomer concentration 1.96 M), and slower polymerization rates are observed when decreasing the catalyst concentration (Table 1). Indeed, while 30 min are required to reach 90% monomer conversion with nTBD/nTMC = 1/20 (Exp. 1, Table 1), 8 h are needed to reach the same monomer conversion with nTBD/nTMC = 1/400 (Exp. 4, Table 1). At the end of each experiment (1–4, Table 1), PTMCs were recovered by filtration after precipitation of the reaction media in diethyl ether and analyzed by ^1^H NMR spectroscopy in CDCl_3_ (Figure 1). In each spectrum, the lack of a peak attributable to end-group protons suggested the formation of PTMC with a cyclic topology and prevented any calculation to determine average molecular weights. Recovered PTMCs were thus characterized by triple SEC in DMF, which provided effective molecular weights ranging from Mn = 14,800 to 33,700 g mol^−1^ and with dispersities less than 1.55 (Table 1).

**Figure 2:**
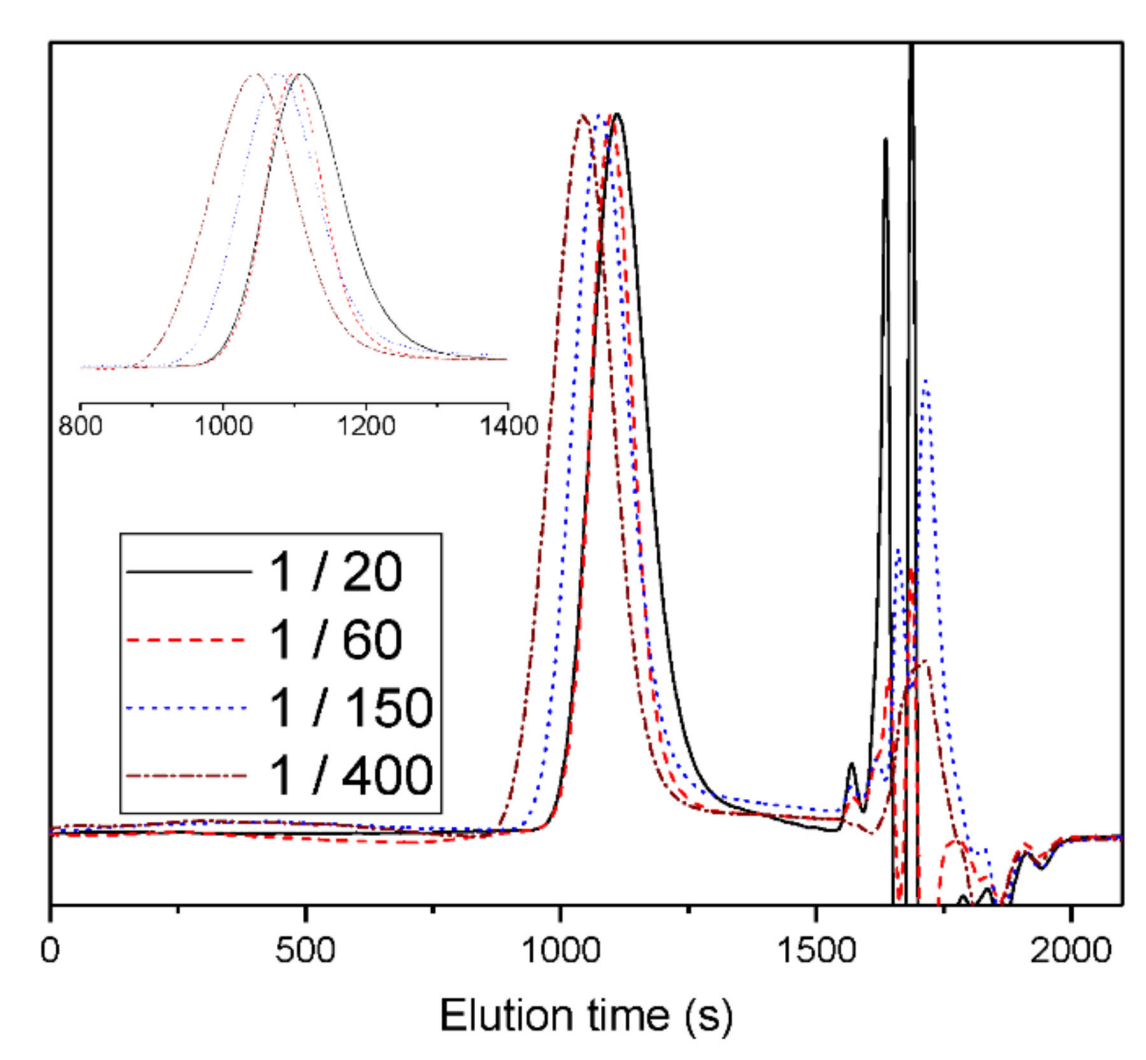
Size exclusion chromatograms of PTMCs from Exp. 1–4, Table 1, prepared by ZROP with a different ratio of nTBD/nTMC (in DMF at 0.8 mL min^−1^).

As previously observed for other ZROP, no clear relationship between the molar ratio n_catalyst_/n_monomer_ and the molecular weight of the formed polymer can be settled, thus preventing any theoretical prediction of the final molecular weight. As discussed in previous studies, the rates of propagation and cyclization have an influence on the molecular weights and depend on the monomer/catalyst couple, thus preventing any prediction of the molecular weight beforehand [32]. Nevertheless, as in other ZROP, it can be observed that a decrease in catalyst loading led to polymers with higher molecular weights (Figure 2).

Besides the influence of the ratio nTBD/nTMC on PTMC molecular weights, we also noticed that molecular weight distributions (Ð) were affected by the duration of the polymerization reaction. Indeed, if the polymerization is not stopped right after reaching high monomer conversion, SEC traces show an enlargement of the molecular weight distribution. This result can be attributed to the decrease in the monomer concentration at the end of the reaction, which naturally leads to the formation of macrocycles with a smaller degree of polymerization.

To confirm the formation of macrocyclic PTMCs during these experiments (Exp. 1–4, Table 1), precipitated polymer samples were also characterized by two complementary techniques: MALDI-TOF mass spectrometry and viscosimetry. Indeed, if macrocyclic PTMCs are present in the samples, MALDI-TOF mass spectra should reveal peaks corresponding to the mass of PTMCs lacking end groups. On the other hand, differences in intrinsic viscosities should be observed between linear and ZROP PTMCs of the same molecular weight. Indeed, the [ɳ]_cyclic_/[ɳ] _linear_ ratio should be less than 1. According to previous studies, this ratio can vary depending on the solvent employed and the monomer unit [33].

**Figure 3:**
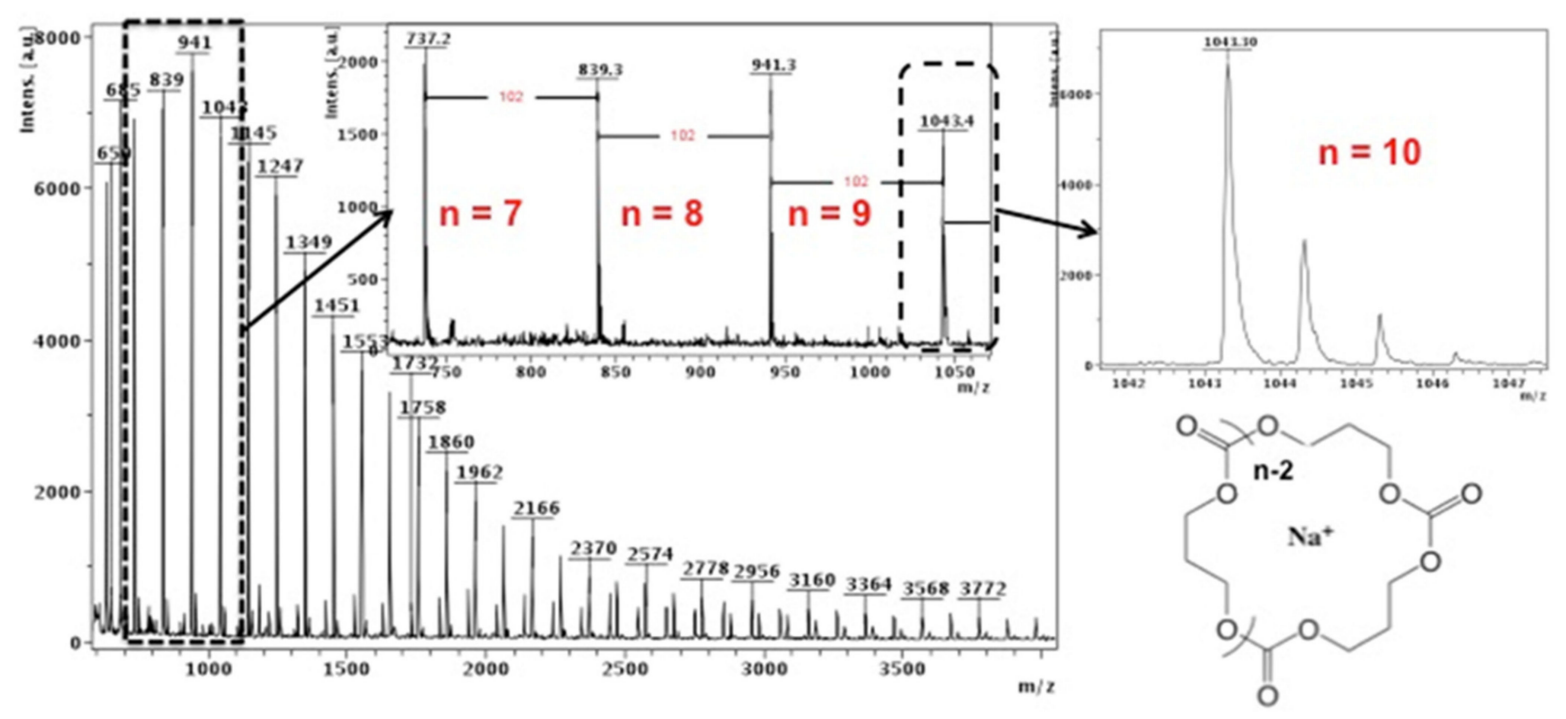
MALDI-TOF mass spectra of PTMC obtained from Exp. 2 (nTBD/nTMC = 1/60) using NaI salt as cationization agent and dithranol as matrix.

As represented in Figure 3, the MALDI-TOF mass spectrum of the precipitated product obtained from Exp. 2, with nTBD/nTMC = 1/60, displays only one population of peaks that are separated by 102 m/z, which corresponds to the molecular weight of one trimethylene carbonate unit.

In addition, the monoisotopic neutral mass of each group of peaks corresponds exactly to the mass of a cyclic PTMC complexed with Na. For example, the peak at 941.3 m/z corresponds to the mass of a Na-complexed PTMC composed of nine monomer units and devoid of chain ends. The MALDI-TOF spectrum of polymer samples obtained with different molar ratios of nTBD/nTMC provide similar results, although different molecular weights have been determined by triple SEC, Supplementary materials (Figure S1). This difference is attributed to the difficulty in ionizing polymer macrocycles but also high molecular weight polymers. From these results, it can thus be concluded that the ZROP of TMC leads to the formation of macrocyclic PTMCs or to a mixture of linear and cyclic PTMCs, the latter being undetectable due to their high molecular weight.

**Figure 4:**
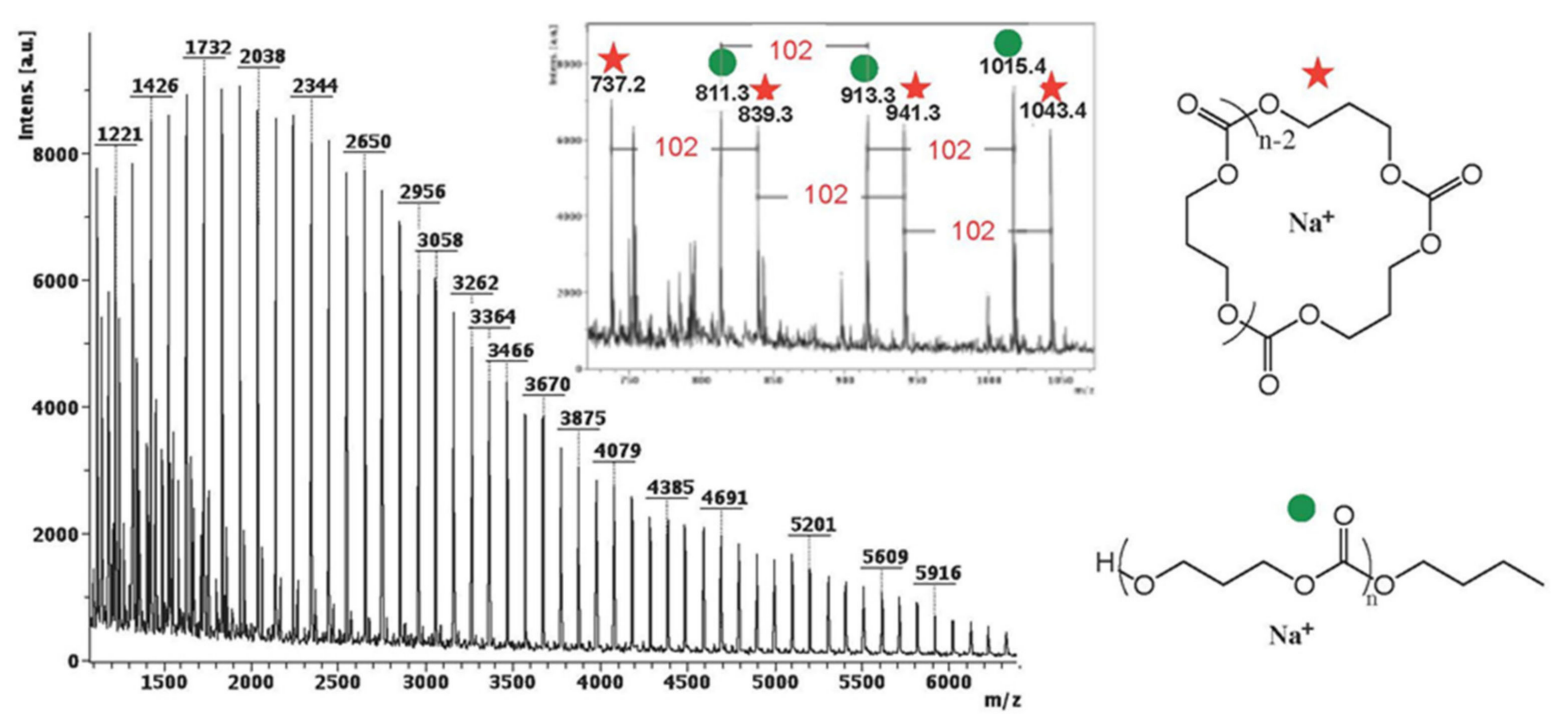
MALDI-TOF mass spectra of PTMC obtained from Exp. 5, Table 1, using NaI salt as cationization agent and dithranol as matrix.

To confirm the presence of PTMC with a cyclic topology by viscosimetry, PTMC samples composed of only linear chains of the same molecular weight as PTMC obtained by ZROP were then synthesized by organocatalyzed ROP. In the first attempt, the polymerization of trimethylene carbonate was thus carried out in THF using TBD as catalyst and butan-1-ol as initiator (Exp. 5, Table 1). After completion, the reaction was quenched with benzoic acid and the polymer recovered by precipitation in diethyl ether. During the reaction, monitoring of the monomer conversion by ^1^H NMR revealed an extremely fast polymerization with a conversion greater than 90% being reached in less than 10 min. A similar observation was made by Meng et al. when using dichloromethane as solvent [34]. As expected, the ^1^H NMR spectra of the precipitated polymer sample (Figure S2a) displayed the characteristic signal of the CH_3_ from butyl chain ends at 0.9 ppm. Using the relative intensity of methyl end groups to those attributed to CH_2_ from monomer units, an average molecular weight of Mn = 11,600 g mol^−1^ could be calculated, very close to the 12,000 g mol^−1^ targeted. However, analysis of the same PTMC sample by triple size exclusion chromatography revealed the presence of two populations overlapping (Figure S2b) and an average molecular weight of 5200 g mol^−1^. This last observation suggests the presence in the PTMC sample of linear chains with butyl chain-ends but also of chains with a cyclic topology. To confirm this hypothesis, analysis by MALDI-TOF mass spectrometry of the PTMC sample from Exp. 5 (Table 1) was thus performed (Figure 4).

As can be observed in Figure 4, the obtained mass spectrum shows the presence of two major populations of peaks. Both of them have signals separated by 102 mass units, corresponding to the trimethylene carbonate monomer unit. Then 74 mass units separate the peaks of each population, which corresponds exactly to the molar mass of butanol. Finally, the respective molar masses of each population of peaks perfectly match the calculated mass of sodium complex of macrocyclic PTMC and of linear chains initiated by butan-1-ol. From these results, it can thus be concluded that the solution polymerization of TMC catalyzed by TBD in the presence of alcohol leads to the formation of both linear and cyclic PTMC. It may be assumed that the balance between the two populations of chains will vary towards the formation of a large majority of linear chains with increasing reaction times or temperatures, since both parameters were found to favor the ROP of macrocyclic carbonates [10]. This may account for the good chain-end fidelity of the TBD-catalyzed ROP of TMC that has been observed during other studies [34]. Nevertheless, too long reaction times should be avoided, as they may also lead to the formation of “small” macrocycles as expected by the Stockmayer theory and as demonstrated by Xie et al. [35].

**Scheme 2:**
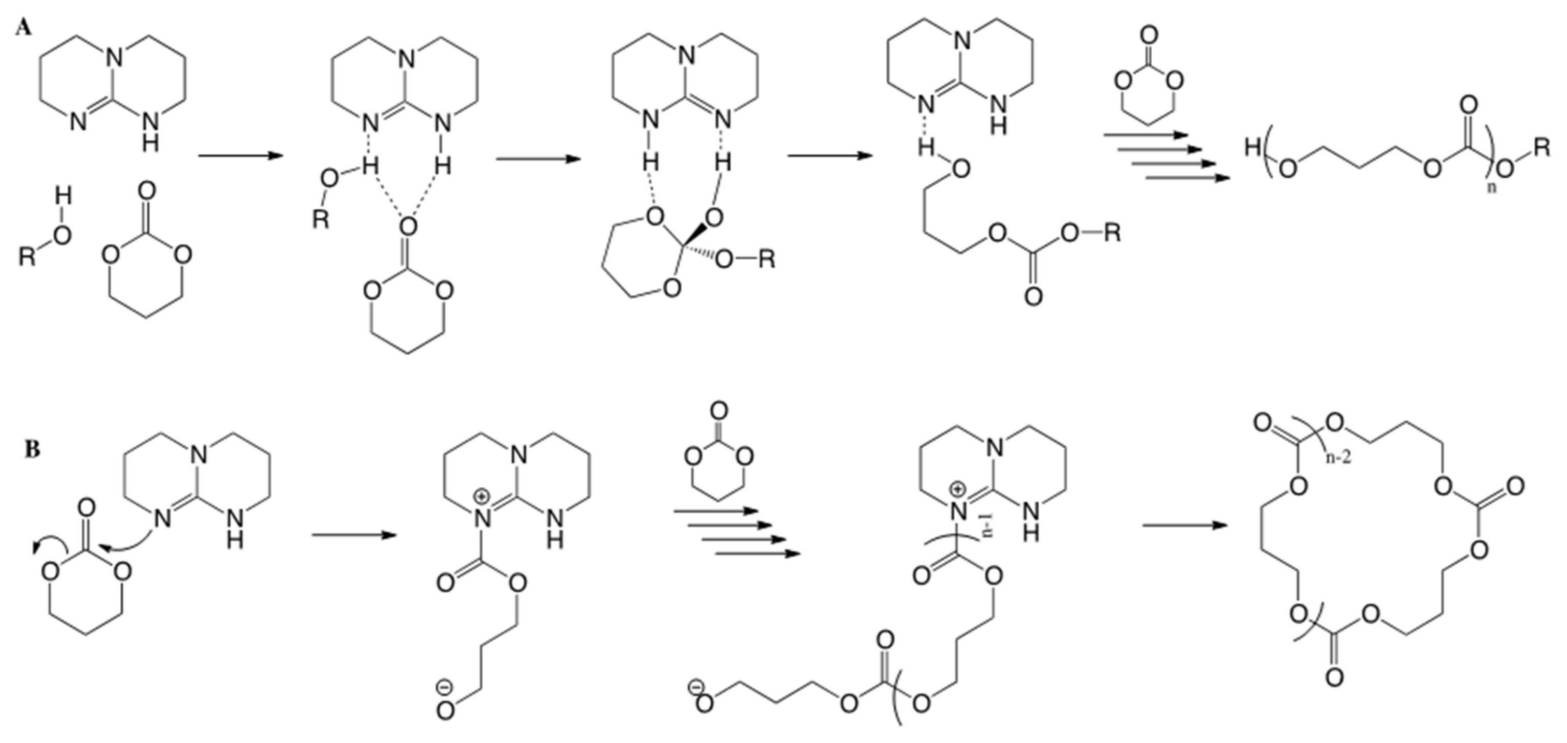
Proposed mechanisms of TMC ROP using TBD as catalyst (**A**) with alcohol or (**B**) without alcohol according to [32,36].

The presence of both linear and cyclic PTMCs was observed in final polymer samples obtained from different experimental conditions as follows: varying the molar ratio nTBD/nROH between 1 and 0.05, using alcohol initiators of different nature (butan-1-ol or benzyl alcohol) or changing the solvent of reaction media by dichloromethane or toluene. The presence of both linear and cyclic structures in the PTMC samples can be explained by the different mechanisms occurring during the ROP of TMC using TBD as catalyst in the presence of an alcohol (Scheme 2).

**Figure 5:**
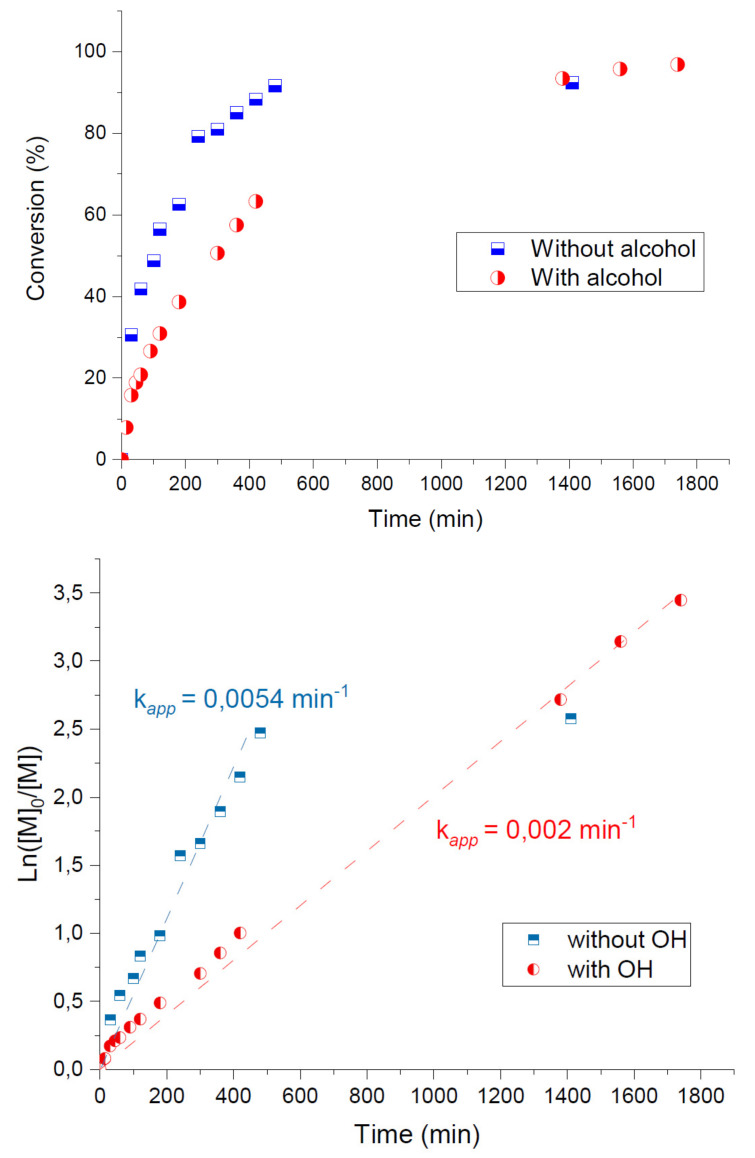
Experimental kinetic data of TMC polymerization catalyzed by TBD in the presence and absence of benzyl alcohol (Exp. 4, Table 1). For the polymerization performed in the presence of alcohol, molar ratios nTBD/nTMC = 1/400 and nTBD/nROH = 1/1 were used.

As previously mentioned, both an activated monomer (AM) mechanism and an activated chain-end (ACE) mechanism have been reported to occur with TBD, leading to linear chains. Indeed, TBD is a bifunctional activator with a basic tertiary amine able to activate the alcohol toward nucleophilic attack and with a hydrogen donating N-H able to activate the carbonyl of TMC. Moreover, a ZROP mechanism can take place when the tertiary amine of TBD behaves as a strong nucleophile and directly attacks the carbonyl of TMC to generate a guanidinium species that stabilizes the tetrahedral intermediate (B, Scheme 2) [36]. In the presence of an alcohol initiator, theoretical studies have indicated the occurrence of a hydrogen-bonding mechanism corresponding to a combination of both AM and ACE mechanisms. In this case, the alcohol and the monomer are simultaneously bonded to the TBD catalyst, leading to the ring opening of the TMC cycle (A, Scheme 2). Without an alcohol initiator, a ZROP mechanism takes place since no nucleophilic species other than the tertiary amine from the catalyst is present in the reaction media. The zwitterionic species formed after nucleophilic attack of the TBD on TMC displaying an alcoholate allows for further attack of TMC and polymer chain growth (B, Scheme 2). Eventually, when no monomer is left or when chain folding allows it, cyclization occurs, which arises from the nucleophilic attack of the alcoholate chain end onto the guanidinium tetrahedral intermediate [32]. Depending on the experimental conditions employed, the decisive point in favor of one or the other mechanisms seems to be the kinetics of TBD–alcohol activation and the kinetics of TBD–monomer zwitterion formation. Thus, in order to determine which mechanisms prevail during the ROP of TMC in the presence of an alcohol, we studied the polymerization kinetics of TMC in the presence and absence of alcohol (Figure 5).

**Scheme 3:**
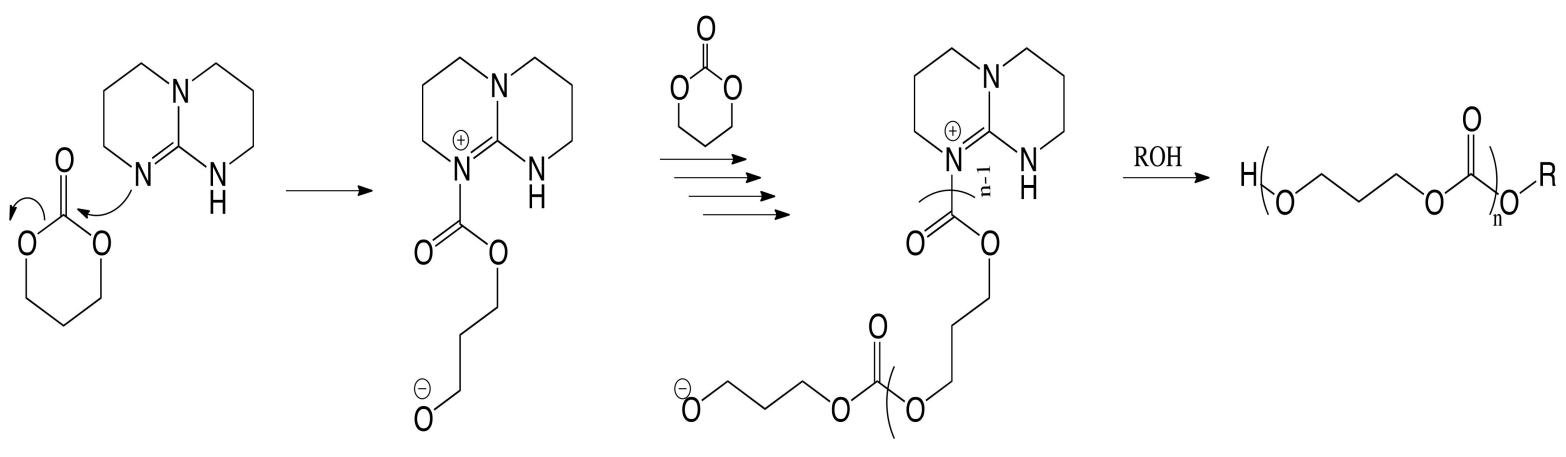
Proposed mechanism of ZROP using TBD as catalyst where alcohol is a terminating agent.

As opposed to the kinetics observed by Waymouth et al. during the polymerization of ε-caprolactone catalyzed by N-heterocyclic carbenes [37], the polymerization of TMC in the absence of alcohol is faster than the polymerization with an alcohol initiator, as evidenced by the apparent polymerization rates values (Figure 5). After 8 h, 90% monomer conversion is reached for the polymerization without alcohol, while in the presence of alcohol, approximately 22 h are required to reach the same value. This has also been observed in the case of a ratio of n_TMC_/n_TBD_ ≈ 160 since 6 h were necessary to achieve 92% conversion in the presence of butanol (with n_TMC_/n_TBD_ = 160; exp 6 of Table 1), while only 90 min were necessary to achieve 95% conversion in the absence of alcohol (with n_TMC_/n_TBD_ = 150; exp 3 of Table 1). It thus seems that the kinetics corresponding to the formation of zwitterionic species from TMC and TBD are faster than the kinetics of alcohol activation by TBD. The zwitterionic pathway thus appears to be the first mechanism occurring during ROP of TMC with TBD, which is consistent with the formation of PTMC macrocycles in the presence of alcohol. With the preferential formation of a zwitterionic intermediate and the presence of macrocyclic species at the end of the polymerization when performing the reaction in the presence of an alcohol, another mechanism can be considered for the formation of the linear PTMC (Scheme 3). It relies on the nucleophilic attack of the alkoxide-type intermediate on the alcoholic proton of the “initiator”. The hydroxyl-terminated PTMC generated can then grow through the hydrogen-bonding pathway.

**Figure 6:**
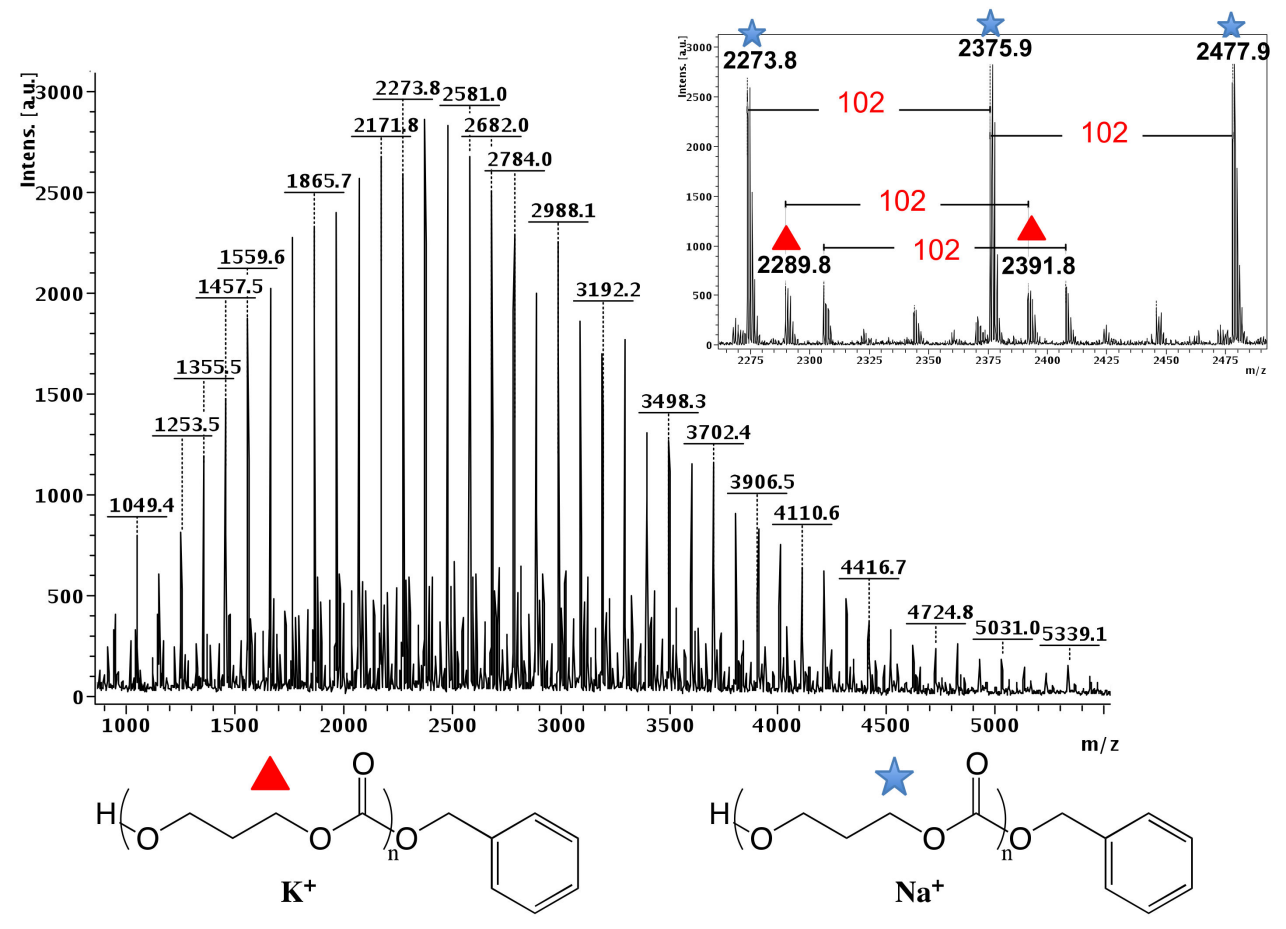
MALDI-TOF mass spectra of linear PTMC obtained from Exp. 7, Table 1.

Formation of PTMC samples containing only linear chains are difficult when using TBD as catalyst, so another organic catalyst must be employed. Among the various organic catalysts for ROP reported in the literature, 4-(dimethylamino) pyridine (DMAP) is less nucleophilic than TBD and has already been employed for the ROP of TMC [38]. We thus employed the same conditions as those reported in the literature to prepare samples devoid of PTMC macrocycles, i.e., using DMAP as catalyst, benzyl alcohol as an initiator and a reaction temperature of 130 °C (Exp. 7 and 8, Table 1). To confirm the presence of only linear chains in the synthesized polymers, the product obtained after precipitation was analyzed by MALDI-TOF mass spectrometry. As can be observed in Figure 6, the mass spectrum corresponding to the polymer sample generated during Exp. 7 (Table 1) shows four populations of peaks. The major population can be attributed to the Na-complexed linear poly(trimethylene carbonate) initiated by benzyl alcohol. Then, a second population corresponds to the K-complexed linear PTMC. Finally, the last two populations of peaks are attributed to the Na and K complexes of linear PTMC that were subjected to transesterification reactions. These transfer reactions are indeed favored at the high temperature of the polymerization. Consequently, using DMAP as catalyst, PTMC samples composed of linear chains only were produced, which allowed us to compare the intrinsic viscosities of PTMC generated by ZROP or those with a linear architecture.

**Figure 7:**
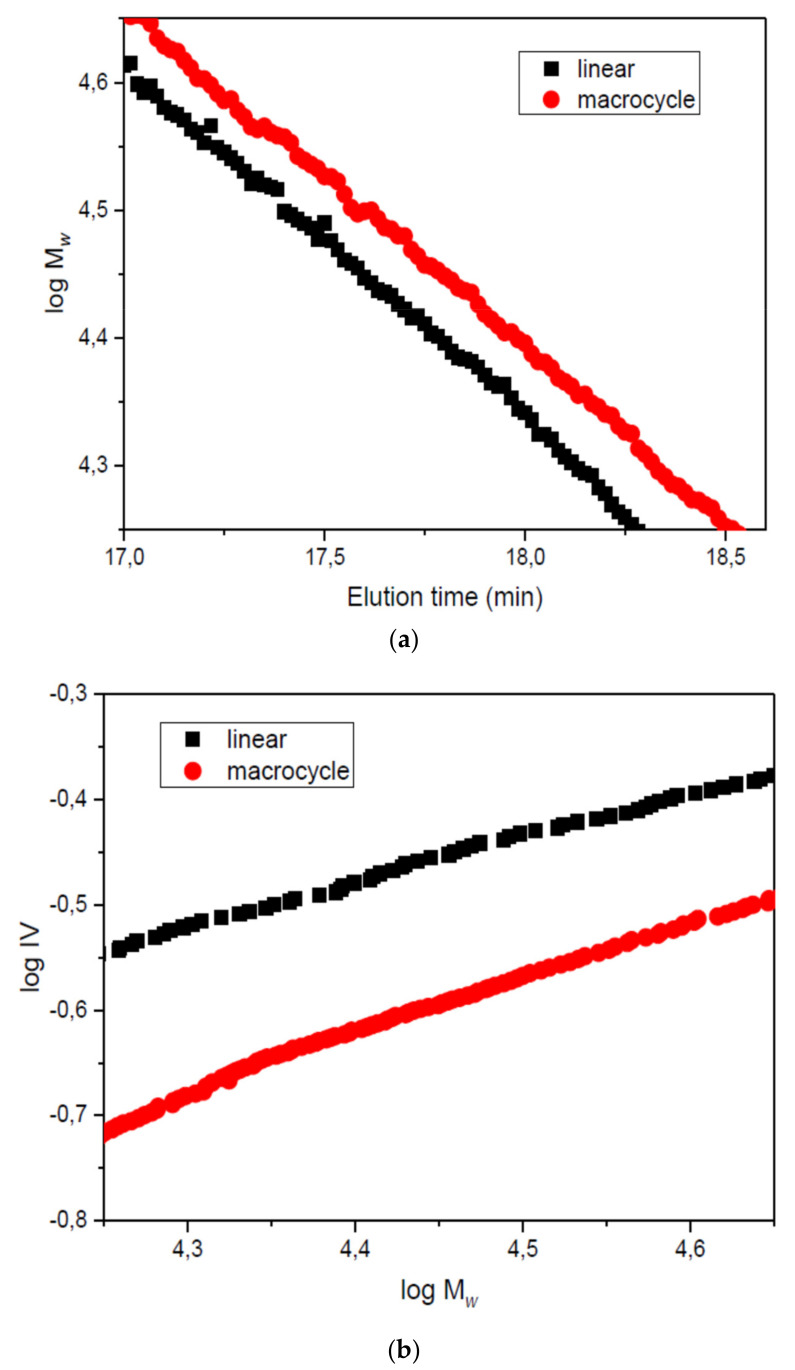
(**a**) Plot of molecular weight logarithm versus elution time; (**b**) double logarithmic plot of intrinsic viscosity (IV) versus molecular weight for PTMC with a molecular weight of 20,500 g mol^−1^ obtained during Exp. 2 and 8 (Table 1).

To determine the ratio of intrinsic viscosities ([η] ZROP/[η] linear), the triple detection SEC equipped with a viscosimeter was employed, as this technique allows the determination of both the effective molecular weight of the polymer and its intrinsic viscosity. To compare the intrinsic viscosity of linear polymers with the same molecular weights as those of the PTMC previously synthesized by ZROP, aliquots from the DMAP-catalyzed ROP of TMC (Exp 7 and 8, Table 1) were withdrawn every 10 min and analyzed by triple SEC. As can be seen in Table 1 (Exp. 7 and 8), two linear polymers with molecular weights very close to PTMC obtained by ZROP from Exp. 1 and 2 were thus obtained (Figure S3). In both cases, the PTMC obtained by ZROP eluted later than the linear PTMCs of the same molecular weight (Figure 7a), providing further evidence of the presence of macrocycles in the PTMC samples obtained by ZROP. Indeed, it is now well established that macrocyclic polymers have smaller hydrodynamic volumes than their linear analogues [28].

Viscosity measurements performed with a viscometer coupled to a light-scattering detector and a refractometer also revealed that PTMCs prepared by ZROP in the absence of alcohol have lower intrinsic viscosities than their linear counterparts of the same molecular weight (Figure 7b). The [η]_ZROP_/[η]_linear_ ratio determined from the Mark–Houwink plot is approximately 0.77, which also suggests the presence of PTMC macrocycles in the polymer samples obtained by ZROP. Indeed, the intrinsic viscosities of the cyclic polymers are lower than those of the linear polymers of the same molecular weight, and the ratio [η]_cyclic_/[η]_linear_ = 0.662 has been determined in theta solvents [33].

## 4. Conclusions

The ring-opening polymerization of TMC catalyzed by TBD in the absence of alcohol proceeds very rapidly, within minutes at room temperature, providing PTMC samples with Mn ranging between 14,800 and 33,700 g.mol^−1^. Characterization of the as-obtained polymer samples by ^1^H NMR, triple detection SEC and MALDI-TOF mass spectrometry provides evidence for the presence of cyclic macromolecules alongside linear ones, which suggests that the alcohol-free TBD-catalyzed ROP of TMC proceeds by a zwitterionic mechanism. This mechanism also appears to occur when the polymerization is performed in the presence of an equimolar ratio of alcohol initiator and TBD, as in addition to linear polymers, PTMC macrocycles are still generated as attested by MALDI-TOF mass spectrometry.

## Figures and Tables

**Table 1:** Characteristics of PTMCs prepared by organocatalyzed ROP of TMC ((TMC) = 1.96 M).

Exp.	n_cata_/n_TMC_	Catalyst	Alcohol	Temp. (°C)	Reaction Time (min)	Conv. (%) ^a^	Mn (g.mol^−1^) ^b^	*Ð* ^b^
**1**	1/20	TBD	without	25	30	98	14,800	1.31
**2**	1/60	TBD	without	25	60	98	20,400	1.19
**3**	1/150	TBD	without	25	90	95	23,100	1.45
**4**	1/400	TBD	without	25	480	92	33,700	1.55
**5**	1/120	TBD	BuOH	25	30	93	5200	1.17
**6**	1/160	TBD	BuOH	25	360	92	7900	1.5
**7**	1/220	DMAP	BnOH	130	140	---	14,600	1.60
**8**	1/440	DMAP	BnOH	130	110	---	20,600	1.40

(^a^) conversion of TMC was calculated comparing the relative intensity of the peak at 4.4 ppm (CH_2_CH_2_CH_2_) of monomer to signal at 4.2 ppm (CH_2_CH_2_CH_2_) of polymer. (^b^) DMF was used as eluent at 0.8 mL min^−1^, and data were calculated using a triple detection system.

## Data Availability

Data sharing not applicable.

## Supplementary Materials

The following are available online at https://www.mdpi.com/article/10.3390/polym13101589/s1. Figure S1: Maldi-Tof mass spectra of PTMC synthesized by zwitterionic ring-opening polymerization with molar ratios of n_TBD_/nTMC = 1/20 (Exp 1, Table 1), 1/60 (Exp 2, Table 1), 1/150 (Exp 3, Table 1), 1/400 (Exp 4, Table 1), Figure S2: (a) ^1^H NMR spectrum in CDCl_3_ and (b) SEC chromatogram in DMF of the PTMC obtained from Exp. 5, Table 1 (n_TMC_/n_TBD_/n_BuOH_ = 120/1/1). Figure S3: Size exclusion chromatograms of PTMC from Exp. 1, 2, 7 and 8 in Table 1 and used to determine the ratio [η] _ZROP_/[η]_linear_ in DMF at 0.8 mL min^−1^.
