# Supplementary material for: Insight into the Alcohol-Free Ring-Opening Polymerization of TMC Catalyzed by TBD
Source: Polymers (Basel). 2021 May 14;13(10):1589. doi: 10.3390/polym13101589 (PMC8156564; doi:10.3390/polym13101589)
Supplement: Supplementary file 1 [file polymers-13-01589-s001.zip › polymers-1188253-supplementary.pdf]

Supplementary information

Synthesis and characterization of trimethylene carbonate-based macrocycles by zwitterionic ring-opening polymerization.

Fabrice Azemar, Olinda Gimello, Julien Pinaud, Jean-Jacques Robin, Sophie Monge*

ICGM, Univ Montpellier, CNRS, ENSCM, Montpellier, France.

E-mail: jean-jacques.robin@umontpellier.fr

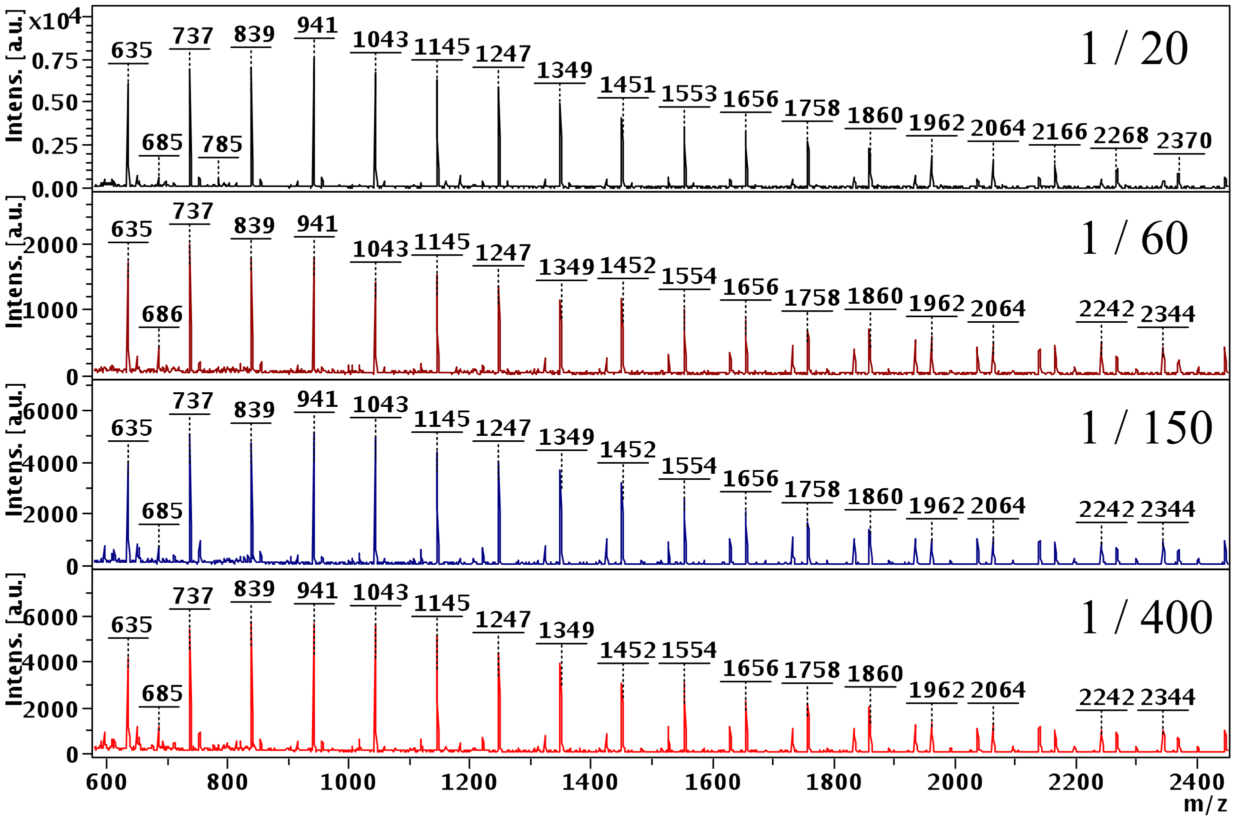

Figure S1. Maldi-Tof mass spectra of PTMC synthesized by zwitterionic ring-opening polymerization with molar ratios of n_{TBD}/nTMC = 1/20 (Exp 1, Table 1), 1/60 (Exp 2, Table 1), 1/150 (Exp 3, Table 1), 1/400 (Exp 4, Table 1).

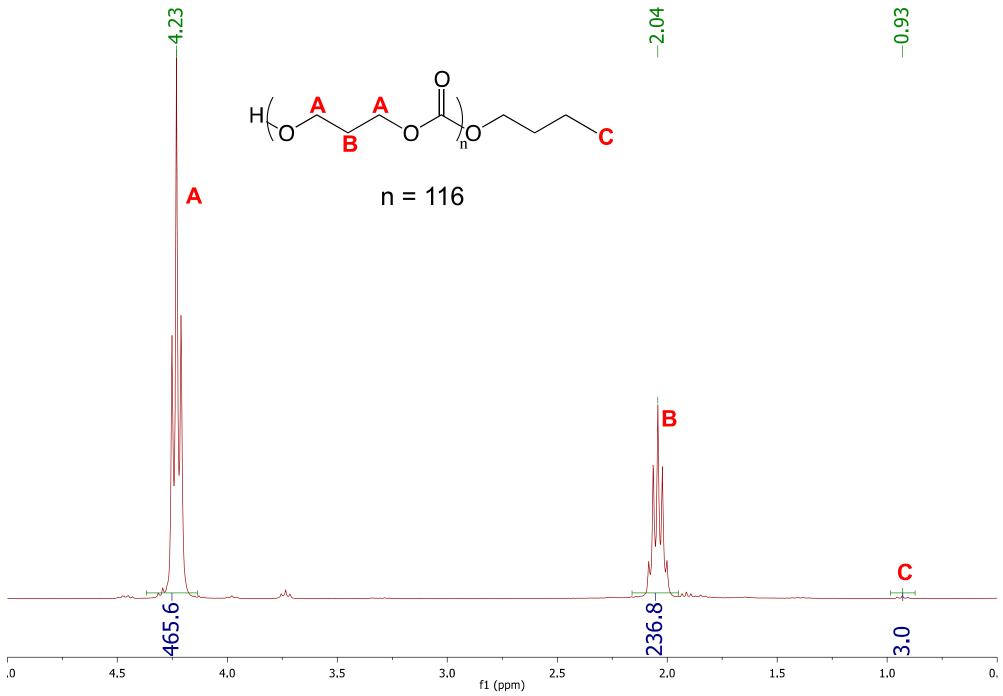

a)

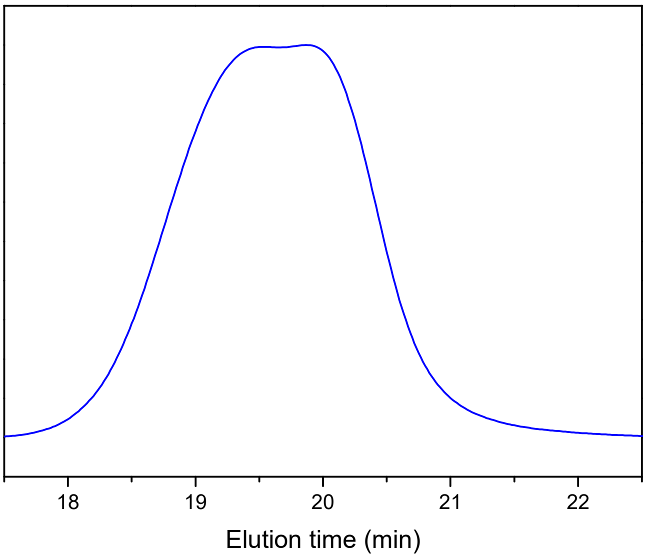

b)

Figure S2. a) ^{1}H NMR spectrum in $CDCl_3$ and b) SEC chromatogram in DMF of the PTMC obtained from Exp. 5, table 1 ($n_{TMC}/n_{TBD}/n_{BuOH}$ = 120/1/1).

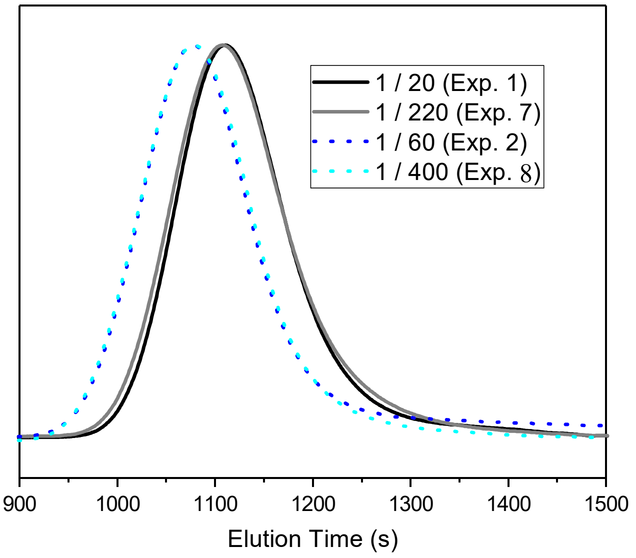

Figure S3. Size exclusion chromatograms of PTMC from Exp. 1, 2, 7 and 8 in table 1 and used to determine the ratio $[\eta]_{ZROP}/[\eta]_{linear}$ in DMF at 0.8 ml min^{-1}.
